# Lumbar percutaneous transforaminal endoscopic discectomy: a retrospective survey on the first 172 adult patients treated in Denmark

**DOI:** 10.1007/s00701-024-06038-6

**Published:** 2024-03-27

**Authors:** Jacob Holmen Terkelsen, Torben Hundsholt, Carsten Reidies Bjarkam

**Affiliations:** 1 School of Medicine and Health, Aalborg University, Selma Lagerløfs Vej 249, 9260 Gistrup, Denmark; 2 Department of Neurosurgery, Aalborg University Hospital, Hobrovej 18-22, 9000 Aalborg, Denmark

**Keywords:** Complications, Discectomy, Endoscopy, Learning curve, Lumbar disk herniation, Spine, Surgery, Transforaminal

## Abstract

**Purpose:**

To evaluate patient demographics, surgery characteristics, and patient-reported clinical outcomes related to the implementation of lumbar PTED in Denmark by surgeons novice to the PTED technique.

**Methods:**

All adult patients treated with a lumbar PTED from our first surgery in October 2020 to December 2021 were included. Data was generated by journal audit and telephone interview.

**Results:**

A total of 172 adult patients underwent lumbar PTED. Surgery duration was a median of 45.0 (35.0–60.0) minutes and patients were discharged a median of 0 (0–1.0) days after. Per operatively one procedure was converted to open microdiscectomy due to profuse bleeding. Post operatively one patient complained of persistent headache (suggestive of a dural tear), two patients developed new L5 paresthesia, and three patients had a newly developed dorsal flexion paresis (suggestive of a root lesion). Sixteen patients did not complete follow-up and 24 (14.0%) underwent reoperation of which 54.2% were due to residual disk material. Among the remaining 132 patients, lower back and leg pain decreased from 7.0 (5.0–8.5) to 2.5 (1.0–4.5) and from 8.0 (6.0–9.1) to 2.0 (0–3.6) at follow-up, respectively (*p* < 0.001). Additionally, 93.4% returned to work and 78.8% used less analgesics. Post hoc analysis comparing the early half of cases with the latter half did not find any significant change in surgery time, complication and reoperation rates, nor in pain relief, return to work, or analgesia use.

**Conclusion:**

Clinical improvements after lumbar PTED performed by surgeons novel to the technique are satisfactory, although the reoperation rate is high, severe complications may occur, and the learning curve can be longer than expected.

## Introduction

Microdiscectomy (MD) has long been regarded as the gold standard in the surgical treatment of lumbar disk herniation (LDH)[31]. However, recently less invasive alternatives such as lumbar percutaneous transforaminal endoscopic discectomy (PTED) have become increasingly popular for selected LDH cases [44]. PTED is performed with a small incision and a lateral spinal approach using an endoscope, minimizing tissue damage, muscle retraction, and bone resection [45]. When performed by a surgeon experienced with the procedure, the indications for lumbar PTED are many [29], and the procedure is quick and associated with less blood loss, faster hospital discharge, less scarring, and equal pain relief compared to MD [5, 18, 19, 26, 30, 37, 43]. However, several challenges exist including LDH at level L5/S1 where the tall iliac crest may make cannula insertion difficult [28], a narrow foraminal space possibly requiring foraminoplasty [29], migrated LDH, and sequestration of the disk [28]. Aside from these challenges, the procedure has a learning curve that may impose an initial increased surgery duration and necessitate more reoperations [3]. The results from implementation of lumbar PTED among surgeons novice to the procedure have been published by several studies prior [1, 6, 7, 15, 16, 25, 33, 34, 39, 46, 48, 49]. However, most of these studies have small study populations and a low patient volume, potentially affecting results and limiting obtainable procedure proficiency [14]. Additionally, no such studies exist from a Danish setting.

Accordingly, the aim of this study was to describe patient demographics, surgery characteristics, and patient-reported clinical outcomes with special focus on complications, reoperations, and clinical efficacy in the first 172 adult patients treated with lumbar PTED at our department.

## Methods

### Study design

This is a retrospective cohort study in which all adult patients treated with a lumbar PTED at our department from the first surgery in October 2020 to the 31st of December 2021 were identified using ICD-10 procedure code KABC07 for percutaneous endoscopic LDH removal.

### Patient selection

Generally, patients are referred for surgical evaluation if they have suffered from persistent lumbar back pain despite relevant conservative treatment for at least 8–12 weeks and have an MRI scan demonstrating a lumbar disk herniation. To secure a proper number of surgical PTED candidates, a special visitation practice was established securing that the referral text and corresponding MRI of all patients initially were seen by one of the two selected PTED surgeons (cherry picking), thereby ensuring that no PTED suitable candidate should be lost to a conventional MD surgeon. The selected PTED candidates would then be clinically evaluated by one of the two selected PTED surgeons in the out-patient clinic and signed up for PTED surgery if history, clinical signs, and MRI were found in alignment. Thus, a good PTED candidate would have one-level disk disease with singular root affection reachable through a large foramen (generally younger patients with high disk space and less foraminal spondylosis), whereas older spondylotic patients with lumbar back pain from multilevel disease would be most unsuitable PTED candidates. Additionally, for patients with L5/S1 herniations, the height of the iliac crest was evaluated on the corresponding MRI or an additionally obtained X-ray.

### The lumbar PTED procedure

The procedures were performed using a Transforaminal Endoscopic Surgical System (joimax GmbH, Karlsruhe, Germany) by two neurosurgeons experienced with spinal surgery, but novice to lumbar PTED. Before performing any procedures alone, the surgeons attended a webinar and participated in hands-on training at established PTED facilities. Furthermore, they received in-house assistance from a surgeon experienced with lumbar PTED during the first three procedures. After this, approximately three procedures with the participation of both surgeons were performed per week.

**Fig. 1:**
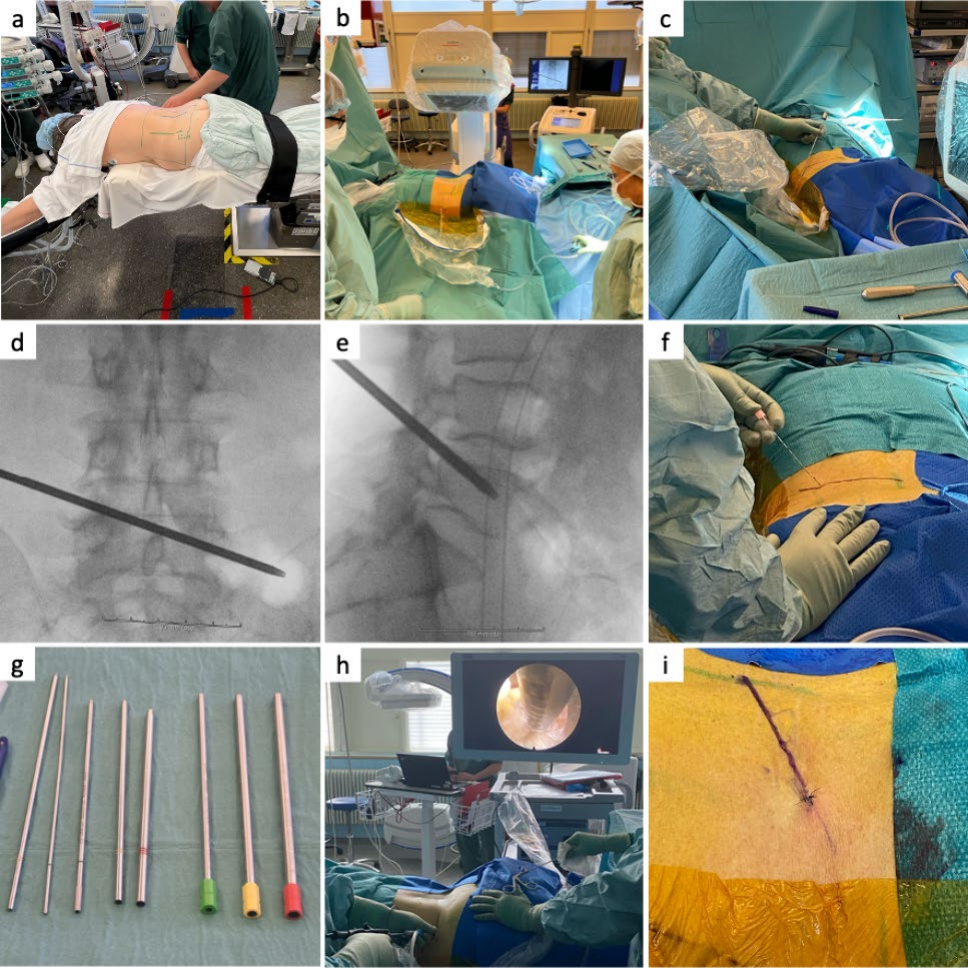
Images of a lumbar percutaneous endoscopic discectomy showing a patient in prone position (**a**); the surgeon, nurse, and instruments setup (**b**); the marking of the anterior–posterior and the lateral line using a steel rod and X-ray (**c**); the marking of the anterior–posterior line (**d**); the marking of the lateral line (**e**); the insertion of the 18-G needle (**f**); the dilatators and reamers used to widen the intervertebral foramen (**g**); the removal of loose tissue and herniated fragments under full endoscopic view (**h**); and the skin closure with a single suture (**i**)

All patients were operated in general anesthesia. The patient was positioned in a prone position with a cushion supporting the breast and a cushion supporting the hip to extend the lumbar spine and increase the foraminal entry (Fig. 1a). The surgeon stands on the side of the pathology, with the scrub nurse and the instruments table positioned beside the surgeon, and the endoscope tower, monitor, and the C-arm positioned opposite the surgeon (Fig. 1b). Access to the herniated disk is gained through the intervertebral foramen from which the nerve root exits. The transforaminal approach aims directly at the medial aspect of the foraminal annular window (Kambin’s triangle). The entry point for L3/L4, L4/L5, and L5/S1 is approximately 8–10, 10–12, and 12–14 cm from the middle of the back, respectively. With the use of X-rays, an AP and a lateral line are marked using a steel rod (Fig. 1c). The AP line is drawn from the upper border of the ipsilateral pedicle to the inferior border of the contralateral pedicle of the caudal vertebra (Fig. 1d [47]). Then, a lateral line which transects the posterior upper corner of the caudal vertebra and the ventral superior articular process is drawn (Fig. 1e [47]). The needle’s entry point is where both these lines meet. Once the entry point is marked, the skin and the trajectory are infiltrated with local anesthetics. An 18-G needle is advanced (Fig. 1f) using X-rays from the entry point to the landing point which is at the upper corner of the caudal vertebra on the lateral view and at the medial pedicle wall on the AP-view simultaneously. A three-step guide wire concept is used to access the herniation. The soft tissue path is gradually dilated under X-ray control, and the foramen is gradually widened using reamers increasing in diameter (Fig. 1g). This provides a root-conserving access corridor to the spinal canal and the herniation. The instruments (guiding rods, guiding tubes, disposable reamers, and reamer ejectors) are color-coded in the logical sequence of a traffic light: green-yellow–red, where the green-marked instruments have the smallest diameter and the red-marked the largest (Fig. 1g). Once the reamer meets bone, it is rotated clockwise to drill. A tubular working channel is introduced, and its opening is directed to the dura. An X-ray is performed to confirm correct positioning of the working channel before introducing the 30° angled endoscope and checking proper positioning of the camera. A pressure regulated pump is used for rinsing with 9% saline. Loose tissue and herniated fragments within the lower foramen and recess are removed using grasper forceps and bipolar cautery under full endoscopic view (Fig. 1h). After evacuating all herniated fragments, an endoscopic check is performed to verify that the affected nerve root has been relieved of pressure and can move freely. The working channel and the endoscope are retracted, and the skin is closed with a single suture (Fig. 1i).

### Data generation

Baseline characteristics were generated from the medical files of each patient and entered in REDCap [20, 21]. Follow-up data was generated by telephone interview at least 6 months after surgery. During the telephone interview, patients were instructed to quantify lower back and leg pain before PTED and at the time of the interview using an 11-point numerical rating scale (NRS) from zero to ten. Zero represents no pain and ten the worst pain imaginable. Patients were also asked about surgery complications including nerve root injury, bleeding, infection, spinal headache, work status, analgesic use, and reoperation. Reoperation was defined as additional surgery on the same level and side of disk as the initial lumbar PTED. Acute reoperations were defined as occurring within the same hospital admission as the initial PTED, while later reoperations were later hospital admissions. The cause of reoperation was determined based on the surgeon’s and the radiologist’s interpretation of the additional MRI scan prior to the reoperation and the course of the clinical manifestations from the initial PTED to the reoperation described in the medical files and in the telephone interview. A herniation reduced in size following surgery combined with no leg pain free period was considered to represent an incomplete decompression case. A herniation increased in size after a leg pain free period was considered to represent a reherniation case. To identify possible reoperations that had not occurred at follow-up, the medical files of each patient were reviewed again at least 1 year after the procedure.

### Data analysis

Patient demographics, surgery characteristics, and number of reoperations were based on all included patients, while only patients who completed the follow-up telephone interview and had not undergone reoperation at follow-up were included in the follow-up data analysis. Additionally, one patient who was involved in a car crash and had additional lower back surgery on an unknown level was excluded. The procedures were described as successful if no reoperation had occurred at the late follow-up. With regard to analysis of leg pain before PTED and at follow-up, only pain in the leg corresponding to the side of the PTED procedure was included. Patients who underwent bilateral PTED were also included by adding the leg pain value from both legs and taking the mean.

### Statistics

Mean and standard deviation (SD) were used to describe normally distributed data, while median and interquartile range (IQR) were used to describe non-normally distributed data. A Wilcoxon matched pairs signed rank test was used to analyze the difference in patient-reported pain before PTED and at follow-up. Pearson’s chi-squared test and Fisher’s exact test were used to analyze the difference in reoperation rates between L4/L5 and L5/S1 procedures and early and late cases. Additionally, Fisher’s exact test was used to analyze the difference in complication rates between L4/L5 and L5/S1 procedures. Data analysis and statistics were performed in RStudio (Posit Software, PBC, Boston, MA, USA).

## Results

**Fig. 2:**
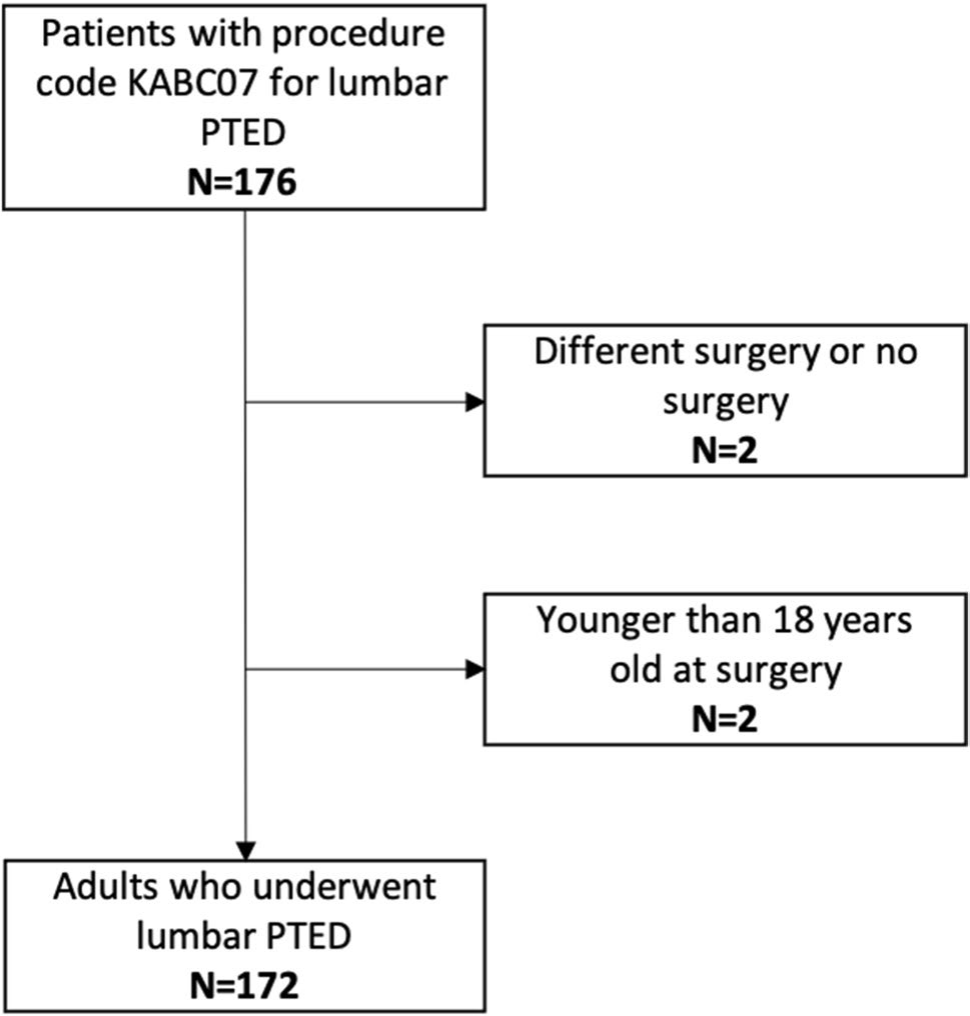
Flow chart of patients included in the study population who underwent lumbar PTED in 2020 and 2021 at our department. Abbreviations: PTED percutaneous transforaminal endoscopic discectomy

A total of 172 adult patients underwent lumbar PTED in 2020 and 2021 at our department (Fig. 2).

The patient demographics and surgery characteristics are seen in Table 1. Additionally, the patients had a median of 12.0 (6.0–22.50) months of lower back and leg pain before surgery, 161 underwent one-level unilateral lumbar PTED, three two-level unilateral, and eight bilateral, and they were discharged a median of 0 (0–1.0) days after surgery; 97 (56.4%) the same day and 63 (36.6%) the following day.

**Table 1:** Demographics and surgery characteristics of patients who underwent lumbar PTED

Patients, *n*	172
Mean (SD) age at surgery, years	51.4 (± 14.2)
Female sex, %	50.9
Previous lower back surgery, %	12.8
Median (IQR) surgery duration^a^, minutes	45.0 (35.0–60.0)
Level of disk operated on^b^, *n*	
L2/L3	4
L3/L4	10
L4/L5	124
L5/S1	45
Prolapse location, %	
Foraminal	34.3
Recess	52.9
Both foraminal and recess	8.7
Extraforaminal	1.2
Not described	2.9
Intraoperative complications, *n* (%)	
Conversion to microdiscectomy	1 (0.58)
Postoperative complications, *n*	
Hematoma	1 (0.58)
Headache	1 (0.58)
New motor disturbances	3 (1.7)
New sensory disturbances	2 (1.2)
Reoperation^c^, *n* (%)	24 (14.0)

^a^Excluding 46 patients with no information on surgery duration. ^b^There were six levels of disk operated on among three surgeries with a two-level unilateral approach, and 16 levels among eight surgeries with a bilateral approach, resulting in 11 more observations than surgeries performed. ^c^Defined as additional surgery after the initial PTED on the same level and side of disk

*n* number, *SD* standard deviation, *IQR* interquartile range, *PTED* percutaneous transforaminal endoscopic discectomy

The total complication rate including both intraoperative and postoperative complications was 4.7% (Table 1). There were no dural tears, nerve root injuries, or postoperative infections reported. However, per operatively one procedure was converted to open microdiscectomy due to profuse bleeding and postoperatively one patient complained of persistent headache (suggestive of a dural tear), two patients had new L5 paresthesia, and three patients had a newly developed dorsal flexion paresis (suggestive of a root lesion). Regarding the cause of the reoperations, 13 (54.2%) were due to residual disk hernia material, nine (37.5%) were due to reherniation, and two (8.3%) were uncertain. Four (16.7%) reoperations were acute within the same admissions as the PTED, and 20 (83.3%) were after discharge.

Fifteen patients failed to complete the follow-up telephone interview, one patient was excluded following a car crash necessitating additional lower back surgery on an unknown level, and 24 underwent reoperation, leaving 132 patients with comparable follow-up data eligible for analysis. At follow-up a median of 12.8 (9.9–15.8) months after surgery compared to before PTED lower back pain decreased from 7.0 (5.0–8.5) to 2.5 (1.0–4.5) (*p* < 0.001) and leg pain decreased from 8.0 (6.0–9.1) to 2.0 (0–3.6) (*p* < 0.001). Additionally, 93.4% of patients actively working before surgery returned to work, and 78.8% used less analgesics (13.6% used the same amount and 7.6% used more).

### Post hoc analysis

Patients who underwent PTED at levels L4/L5 and L5/S1 were the largest groups in this study. Both groups had similar ages at surgery, sex, previous lower back surgery rates, surgery durations, complication rates, days hospitalized after surgery, and reoperation rates. Leg pain reduction at follow-up in the L4/L5 group was 6.5 points on the NRS scale, compared to 4.5 in the L5/S1 group (*p* = 0.04, not statistically significant when adjusting for multiple comparisons).

These two largest groups, as well as all other patients, were further divided into 86 early and 86 late cases based on date of surgery. Even though the reoperation rate was 16.3% among early cases and 11.6% among late cases, the difference was statistically insignificant alongside differences in surgery duration which remained at a median of 45.0 min and in complication rates which were 4.7% for both groups (Table 2).

**Table 2:** Surgery duration, total complication rate, and reoperation rate among the first 86 cases and the last 86 cases

	Early cases	Late cases	*p* value
Patients, *n*	86	86	
Surgery duration^a^, median (IQR)	45.0 (35.0–60.0)	45.0 (35.0–60.0)	0.70
Total complication rate, %	4.7	4.7	1.0
Reoperation^b^ rate, %	16.3	11.6	0.38

^a^Excluding 19 and 27 patients among early and late cases, respectively, with no information on surgery duration. ^b^Defined as additional surgery after the initial PTED on the same level and side of disk

*n* number, *IQR* interquartile range

Similarly for the 132 patients reached by telephone, we were not able to find any significant change in lower back and leg pain reductions, return-to-work rates, or analgesic use between the early and the late half of surgeries (Table 3).

**Table 3:** Pain reduction, return-to-work rate, and analgesic use among the first 66 cases and the last 66 cases with comparable follow-up data

	Early cases	Late cases	*p* value
Patients, *n*	66	66	
Lower back pain reduction, median (IQR)	3.8 (1.5–6.0)	3.5 (1.5–5.9)	0.83
Leg pain reduction, median (IQR)	5.0 (2.6–7.5)	5.0 (2.5–7.9)	0.84
Return-to-work rate^a^, %	84.1	89.1	0.55
Less analgesic use, %	74.2	83.3	0.20

^a^Including patients working full time, part time, or on sick leave leading up to surgery. Patients excluded were either retired or not in job leading up to surgery

*n* number, *IQR* interquartile range

## Discussion and conclusion

Although excellent papers on RCTs comparing PTED versus MD [8, 17, 19] and expert papers on advanced PTED applications [12, 13, 29] are widely available, the scientific rationale and merits of current paper are that it give a detailed account of how the PTED technique was implemented and performed by two surgeons initially novice to the procedure, achieving good clinical efficacy, although the reoperation rate was high (Table 1), severe complications were encountered (Table 1), and the learning curve longer than expected (post hoc analysis and Table 2 and 3). Thus, we expect these results to be useful and of interest to others engaging in the PTED technique for the first time.

### Issues to consider before lumbar PTED is implemented in your department

For neurosurgeons doing conventional open spine surgery, the PTED technique is new and demanding concerning patient selection, equipment, surgery, and anatomical landmarks. To overcome these obstacles, you need a proper surgical volume and a dedicated staff and supportive department. Although PTED in the long run may be as cost-effective as MD [17], financing of equipment and training will initially need to be resolved.

To gain expertise and keep a proper learning curve, it is wise to allocate the procedures to selected surgeons and staff. Thus, it was from the start decided by the departmental head that the procedure should be restricted to two senior spine surgeons, and that they would be allowed to pick suitable PTED patients (see “Methods”) before they were seen by anyone else.

The selected surgeons attended several meetings and training courses focusing on PTED not only to learn the technique but also to meet and try equipment provided by different firms (we strongly recommend this step). As a result, more than 2 years passed before the PTED equipment finally was purchased and installed in our department.

During this process important relations were established to experienced PTED surgeons and equipment providers. Thus, the first three procedures in our department were performed on the same day with the participation of an experienced PTED surgeon and the local equipment provider ensuring that all surgical and technical up-start problems could be solved on-site. Although all the remaining surgeries have been performed without supervision, we have greatly benefitted from knowledge exchange within the PTED community and continued participation in PTED training courses for discussions on difficult cases and practical tips and tricks.

### Patient volume, complications, reoperations, and clinical efficacy

The frequency of approximately three procedures per week resulted in a greater patient volume (172 patients in 14 months) than other studies on the implementation of lumbar PTED [1, 6, 7, 15, 16, 25, 33, 34, 39, 46, 48, 49], theoretically creating a better foundation for procedural proficiency.

Both intra- and postoperative complications were few, with a total complication rate of 4.7% (Table 1). Similar studies including surgeons initially novice to lumbar PTED report a total complication rate varying from zero to 16.7% [1, 6, 15, 16, 25, 33, 34, 46, 49]. Additionally, our total complication rate is similar to the 4.6% for lumbar PTED and lower than the 15.9% for MD, reported by a recent systematic review and meta-analysis including surgeons experienced with both procedures [18]. One of the more serious possible intraoperative complications is nerve root injury [52]. While there were no reports of intraoperative nerve root injuries, two and three patients experienced new sensory (L5 dermatome paresthesia) and motor (dorsal flexion paresis) disturbances following surgery, respectively, potentially indicative of intraoperative nerve root damage. This putative nerve root injury rate of 2.9% is similar to the 1.0–8.9% reported by other studies on lumbar PTED [11, 52] and reflects in our view the risk of damage to the exiting nerve root when the endoscope is passed through the intervertebral foramen and the restricted overview of the root location provided by the endoscope view. We therefore recommend newcomers to the PTED technique to avoid patients with a narrow intervertebral foramen caused by bony degenerative changes, low disk height (elderly spondylotic patients), and PTED unfriendly anatomy (the L5/S1 foramen is often quite narrow).

The total reoperation rate in this study is 14.0%, similar to 14.6% reported by a systematic review and meta-analysis including studies with surgeons initially novice to lumbar PTED [3], but higher than 2–10% among surgeons experienced with the procedure [18]. This suggests that the reoperation rate may decrease as experience increases. Regarding the cause of reoperation in this study, 7.6% of all patients underwent reoperation due to incomplete decompression and 5.2% due to reherniation. Incomplete decompression is thought to be more common among surgeons novice to the procedure [12, 50], partly due a suboptimal final location of the working channel [12], and failure to recognize the end point of the procedure described as complete removal of all fragments sequestered from the maternal disk and free mobilization of neural tissue [2]. Supporting this, the rate of patients undergoing reoperation due to incomplete decompression among surgeons experienced with the procedure is lower, ranging from 2.8 to 5.0% [8, 12, 42].

Among the 132 patients with successful lumbar PTED and complete follow-up data, we found a significant clinically relevant [40] improvement in leg and lower back pain at follow-up among successful procedures of 6.0 and 4.5 points on a NRS, respectively. This is in concordance with other studies on lumbar PTED [38] and MD [41], assuming that NRS and VAS correspond [23]. Additionally, among less subjective and more indirect measurements of pain 93.4% of those working before surgery returned to work at follow-up in concordance with literature [38], and 78.8% used less analgesics. No studies reporting analgesic use both before lumbar PTED and at follow-up were identified, but Gadjradj et al. also report decreasing analgesic use over time, from 2 weeks after surgery to 6 and 12 months after both lumbar PTED and MD [19]. Even though reservations should be made when comparing our results to studies on lumbar MD, partly due to our patient selection, the clinical efficacy found in this study compares well with lumbar MD, in line with a recent randomized controlled trial finding lumbar PTED non-inferior to lumbar MD [19].

### Advantages to lumbar PTED

Throughout performing the procedures and conducting this study, several advantages to lumbar PTED were identified. The procedure is minimally invasive as (1) the introduction of the working channel only requires a small entry that can be closed by a single suture, (2) the surgical duration is short (45.0 min) which is associated with fewer intraoperative complications [9], and (3) the patients can be discharged quickly (56.4% the same and 36.6% the following day). Additionally, lumbar PTED can be performed using local anesthesia only [19]. Furthermore, lumbar PTED is procedurally advantageous when it comes to (4) foraminal herniations as it bypasses the need of facet joint removal for MD that can cause spinal instability necessitating lumbar fusion surgery with increased tissue trauma, surgery duration, and risk of adjacent disk disease [51]. It should be noted that foraminal (and extraforaminal) herniations are described as more difficult to remove than paramedian herniations [29], as they require more maneuvering of the endoscope outside the intervertebral foramen to continuously visualize the herniation. Lumbar PTED is also well suited for (5) revision surgery after MD as the transforaminal route avoids the impact of previous scar tissue [29], and (6) obese patients, as the endoscope is just placed deeper compared to MD where a wider access associated with an increased risk of surgical site infections [36] is needed.

### Disadvantages to lumbar PTED

We also became aware of disadvantages to lumbar PTED that should be respected. Firstly, the reported steep learning curve of lumbar PTED [27] is associated with an initial increased surgery duration [1, 3, 15, 16, 25, 33, 34, 39, 46, 49] and reoperation rate [3, 48]. Although these are reported to improve significantly after 10–40 cases [1, 3, 15, 16, 25, 33, 34, 39, 46, 48, 49], no results improved significantly among the 86 early to 86 late cases including surgery duration (indifferent at 45.0 min) and reoperation rate (16.3 to 11.6%, *p* = 0.38) (Table 2), just as we saw no significant change in pain reduction, return to work, or analgesia use among the early and late half of the 132 telephone interviewed patients (Table 3), indicating a more gradual and longer learning curve. Secondly, revision lumbar PTED following previous PTED on the same level and side is disadvantageous the same way revision MD following previous MD is complicated by scar tissue [35]. Accordingly, most (18/24) of our reoperations were performed as open MD. Lastly, herniations at level L5/S1 can prove challenging to remove due to the iliac crest, the inclination of the level, and the facet joint diminishing the foraminal entry [12, 13]. These factors force a steeper trajectory angle of the working channel towards the intervertebral foramen, resulting in a final location of the working channel further away from the herniation [12, 13], which may lead to insufficient removal of the herniation and affect the success rate [12]. This may be why we found a possibly clinically relevant [40] smaller reduction in leg pain with 4.5 points at level L5/S1 compared to 6.5 points at L4/L5 (*p* = 0.04), which however, was not statistically significant when adjusting for multiple comparisons.

### Patient selection suitable for novice PTED surgeons

As evident by the above advantages and disadvantages of lumbar PTED, adequate patient selection is important to the success of lumbar PTED [3]. Recently, Kotheeranurak et al. proposed a patient selection protocol stating that PTED is suited for (1) foraminal and extraforaminal herniations at any lumbar level and (2) central and paramedian herniations at levels L1/L2 to L3/L4, and levels L4/L5 and L5/S1 without high-grade herniation migration or hindrance of the iliac crest [32]. However, this protocol is based on experienced PTED surgeons, and no protocol for novice PTED surgeons exists. Based on our experiences from performing the surgeries and conducting this study, we believe the learning curve of novice PTED surgeons will benefit from being even more select with early cases by excluding herniations at L5/S1 and prioritizing paramedian over foraminal and extraforaminal herniations while sustaining a high patient volume.

### Limitations

Firstly, this study is retrospective with preoperative subjective measurements generated through a telephone interview a median of 12.8 (9.9–15.8) months after surgery, introducing recall bias that may skew results [4]. Secondly, the use of a telephone interview to generate follow-up data may introduce recency bias (responding in favor of what is latest said) [10], and minor variabilities in the way questions are asked which can affect responses [10]. Thirdly, due to 15 patients not completing the telephone interview, one patient being involved in a car crash, and 24 patients undergoing reoperation, only 132 patients (76.7% of patients in this study) were eligible for follow-up data analysis, potentially affecting its validity [24]. Finally, the study population is specifically selected for lumbar PTED making a direct comparison to results from lumbar MD weak. The results should instead be viewed as experiences with the implementation of lumbar PTED at a new department with surgeons initially novice to the procedure.

### Conclusion

Successful introduction of lumbar PTED among surgeons novice to the procedure should be preceded by theoretical and practical hands-on courses, and the initial surgeries should be performed under the surveillance of an experienced PTED surgeon. Careful patient selection is mandatory, and problematic levels (L5/S1) and problematic hernia locations (extraforaminal, foraminal) should be avoided until proper experience has been gained. To facilitate the learning curve, it should be ensured that the involved surgeons have a continuous high volume of procedures and have the possibility to perform the procedures together. If these precautions are met clinical improvements after lumbar PTED by surgeons novice to the procedure will be satisfactory, although the reoperation rate initially should be expected to be higher than seen with MD, just as severe complications may occur, and the learning curve can be longer than expected.

## Data Availability

Data access can be granted upon request to corresponding author.

## Abbreviations

IQR: Interquartile range
LDH: Lumbar disk herniation
MD: Microdiscectomy
NRS: Numerical rating scale
PTED: Percutaneous transforaminal endoscopic discectomy
SD: Standard deviation

## Code availability

Can be provided upon request to corresponding author.
